# Genome editing in the mushroom-forming basidiomycete *Coprinopsis cinerea*, optimized by a high-throughput transformation system

**DOI:** 10.1038/s41598-017-00883-5

**Published:** 2017-04-28

**Authors:** Shigeo S. Sugano, Hiroko Suzuki, Eisuke Shimokita, Hirofumi Chiba, Sumihare Noji, Yuriko Osakabe, Keishi Osakabe

**Affiliations:** 1 Center for Collaboration among Agriculture, Industry, and Commerce, Tokushima University, Tokushima, Japan; 2 Tokushima Prefectural Agriculture, Forestry and Fisheries Technology Support Center, Tokushima, Japan; 3 Faculty of Bioscience and Bioindustry, Tokushima University, Tokushima, Japan

## Abstract

Mushroom-forming basidiomycetes produce a wide range of metabolites and have great value not only as food but also as an important global natural resource. Here, we demonstrate CRISPR/Cas9-based genome editing in the model species *Coprinopsis cinerea*. Using a high-throughput reporter assay with cryopreserved protoplasts, we identified a novel promoter, *CcDED1*_*pro*_, with seven times stronger activity in this assay than the conventional promoter *GPD2*. To develop highly efficient genome editing using CRISPR/Cas9 in *C. cinerea*, we used the *CcDED1*_*pro*_ to express Cas9 and a *U6-snRNA* promoter from *C*. *cinerea* to express *gRNA*. Finally, CRISPR/Cas9-mediated *GFP* mutagenesis was performed in a stable *GFP* expression line. Individual genome-edited lines were isolated, and loss of GFP function was detected in hyphae and fruiting body primordia. This novel method of high-throughput CRISPR/Cas9-based genome editing using cryopreserved protoplasts should be a powerful tool in the study of edible mushrooms.

## Introduction

Mushroom-forming basidiomycetes are unique in that they develop a three-dimensional organized fruiting body useful for the production of a wide range of secondary metabolites as well as supplying food globally. *Coprinopsis cinerea* is a model basidiomycete species commonly used to study fruiting body development^1, 2^ and has been screened for functional genes regulating fruiting body development^3^. Since meiosis in *C. cinerea* progresses synchronously during spore production^1^, *C. cinerea* has also been used as a model in molecular and genetic studies of meiosis. Recently, transcriptome studies of fruiting body development have been aided by deep sequencing methods^4, 5^. Dissection of the genetic pathways involved in these biological processes has been revealed by these approaches, as well as the identification of some novel promoters. To characterize these genes, it is important to establish and validate their functions *in vivo*. Accordingly, high-throughput transformation and scalable systems for gene perturbation are worth developing in these species to further research into the genetics of the mushroom-forming basidiomycetes.

Genome engineering tools, including high throughput transformation systems, are relatively less well developed in mushroom-forming basidiomycetes than in other model filamentous fungi. In *C. cinerea*, gene targeting methods for application to genetic studies have been developed using Δ*ku70* or Δ*lig4* mutants^6^; however, high-throughput reverse genetics and appropriate reporter assays remain to be established^7^. Although a PEG-based transformation system using oidia of *C. cinerea* has been available for many years^8^, preparation of protoplasts from *C. cinerea* oidia is relatively laborious. In some plant pathogenic fungi, protoplasts can be cryopreserved^9^, which makes it possible to perform a number of transformations with one batch of protoplasts^10^. To our knowledge, there is a report which described cryopreservation of protoplast of basidiomycete^11^, however, assessments of cryopreservation buffers and preservation periods remain matters of research.

Genome editing methods are now commonly used to modify genomes *in vivo* ^12^. Among the more recently emerging technologies are artificially designed nucleases targeting genes of interest that are introduced into cells to induce error-prone double strand breaks (DSBs) in target sequences^12^. Currently, the most widespread genome editing technology is the clustered regularly interspaced short palindromic repeat (CRISPR)/CRISPR-associated protein-9 nuclease (Cas9) system, which consists of two components: Cas9 nuclease, and a guide RNA (gRNA) that targets the genome sequence of interest^13^. A number of customizations of the CRISPR/Cas9 system have been introduced for model organisms. The CRISPR/Cas9 system has been developed in various fungi, such as *Neurospora crassa* ^14^, *Candida albicans* ^15^, *Pyricularia oryzae* ^16^, *Trichoderma reesei* ^17^, *Aspergillus fumigatus* ^18^ and *Ustilago maydis* ^19^. However, there are no published reports of the establishment of CRISPR/Cas9 in mushroom-forming basidiomycetes.

The present study demonstrated CRISPR/Cas9-based genome editing and high-throughput transformation methods in the mushroom-forming basidiomycete *C. cinerea*. CRISPR/Cas9 customization to *C. cinerea* was achieved via identification of a novel, constitutively active, promoter screened from a high-throughput transformation method using cryopreserved protoplasts. We developed a novel luciferase assay that takes only 1 day to measure promoter activities in *C. cinerea* protoplasts. This high-throughput transformation and the CRISPR/Cas9 system customized for mushroom-forming basidiomycetes could contribute to the acceleration of genetic studies and molecular breeding in these fungi.

## Results

### Cryopreservation of protoplasts with sorbitol and DMSO buffer

**Figure 1:**
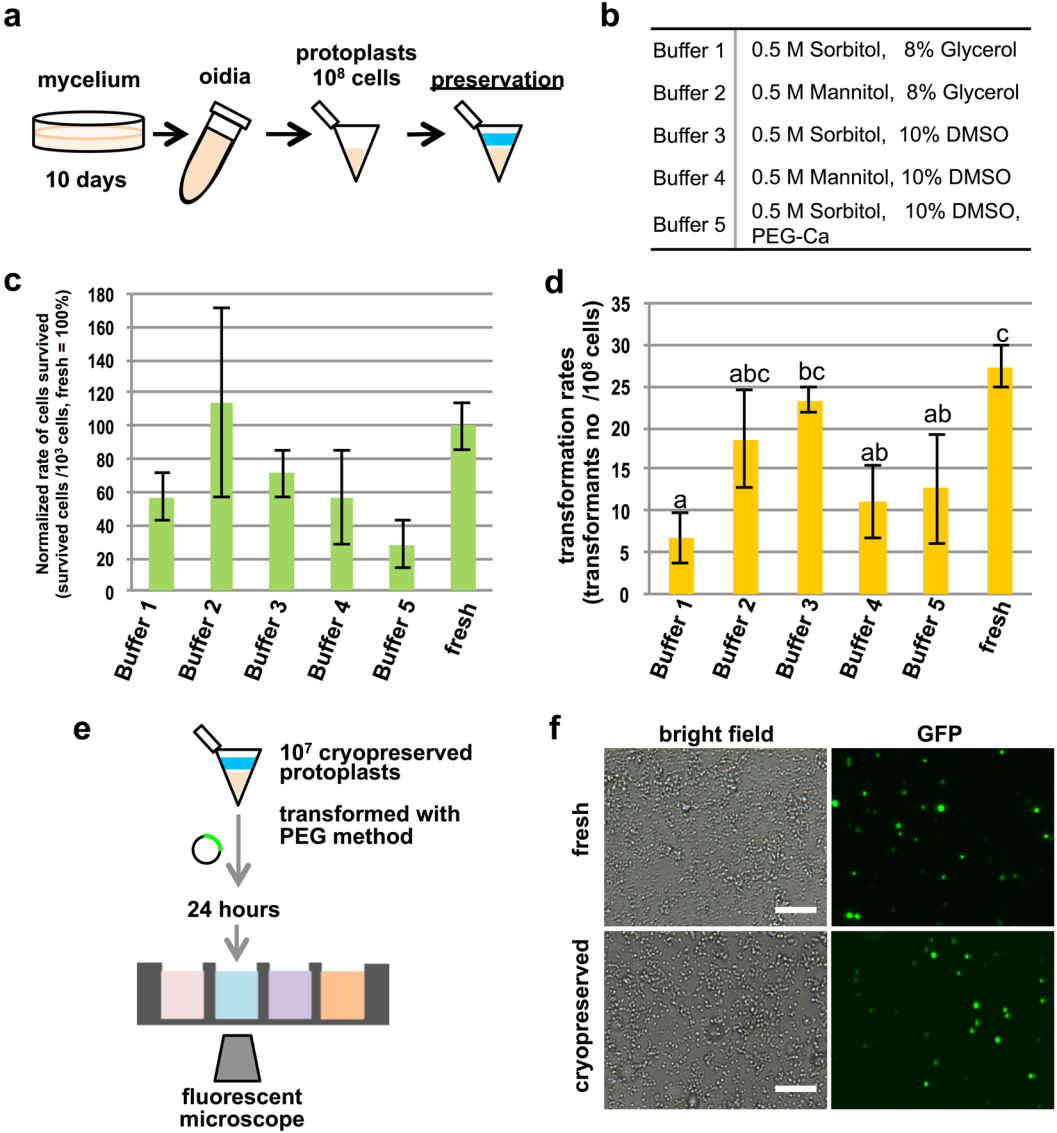
Cryopreservation of protoplasts of *C. cinerea*. Evaluation of protoplasts stored in different buffers at −80 °C for 3 weeks. (**a**) Schematic image of the protoplast preservation experiments. (**b**) The buffers used for cryopreservation of protoplasts. (**c**) Survival rates defined by the number of surviving cells per 10^3^ preserved cells. Survival rate of fresh protoplasts was defined as 100%. (**d**) Transformation rates defined by the number of hygromycin-B resistant colonies in 10^8^ preserved cells. Error bars in (**b**) and (**c**) show S.D. n = 3; bar plots with the same lower case characters were statistically significant groups as tested by Turkey’s HSD test (*P* < 0.05). (**e**) Schematic image of the transient expression assay using preserved protoplasts which were stored at −80 °C with buffer 3 for three weeks. (**f**) Fluorescent images of protoplasts 24 hrs after transformation. Fresh and cryopreserved protoplasts were compared. Scale bar: 50 µm.

To establish a concise procedure for conventional PEG-based transformation in *C. cinerea*, we first developed a new method for protoplast preservation. *C. cinerea* protoplasts were preserved at −80 °C after preparation in various buffers (Fig. 1a,b). We modified the MM buffer [0.5 M mannitol and malate buffer (pH 5.5)^8^] commonly used for protoplast preparation in *C. cinerea*, by using sorbitol as an osmotic stabilizer instead of mannitol. To inhibit the growth of ice crystals, DMSO, glycerol, and PEG were tested (Fig. 1b). Protoplasts in five types of buffer (Fig. 1b) were preserved at −80 °C for 3 weeks, and were then analyzed by measuring both survival and transformation rates (Fig. 1c,d). Protoplasts preserved with buffer containing 0.5 M sorbitol and 10% DMSO showed survival and transformation rates comparable to those of freshly prepared protoplasts. Notably, transformants could be obtained even using protoplasts preserved in the buffer for one year (Supplementary Fig. 1). We also tested the effect of the rate of cooling on the transformation rate of preserved protoplasts. When the protoplasts were cooled at a rate of −1 °C/min, their transformation competency was higher than when they were cooled immediately to −80 °C (Supplementary Fig. 2). The transformation rates in independent trials showed different values (Fig. 1d, Supplementary Fig. 2), however, the transformation rates were affected by the slight difference of condition of the fungal cells when oidia and protoplasts were prepared (data not shown).

Using protoplasts cooled slowly in cryopreservation buffer [0.5 M sorbitol and 10% DMSO (pH 5.5)], we next checked the feasibility of transient transformation. Cryopreserved protoplast cells (10^7^) were transformed by the PEG method with a plasmid harboring the *GFP* gene driven by the *glycerol-3-phosphate dehydrogenase 2 promoter* from *Agaricus bisporus* (*AbGPD2*_*pro*_)^7^. The transformed protoplasts were transferred directly to a 96-well glass-bottom dish and inspected under a fluorescence microscope after 24 hours (Fig. 1e). The cryopreserved protoplasts displayed clear GFP fluorescence, with transformation rates comparable to those of fresh protoplasts (Fig. 1f), indicating that protoplasts prepared with buffer including 0.5 M sorbitol and 10% DMSO could be stored at −80 °C without any apparent reduction in transformation competency. We thus used cryopreserved protoplasts for all subsequent experiments.

### NanoLuc luciferase assay in *C. cinerea*

**Figure 2:**
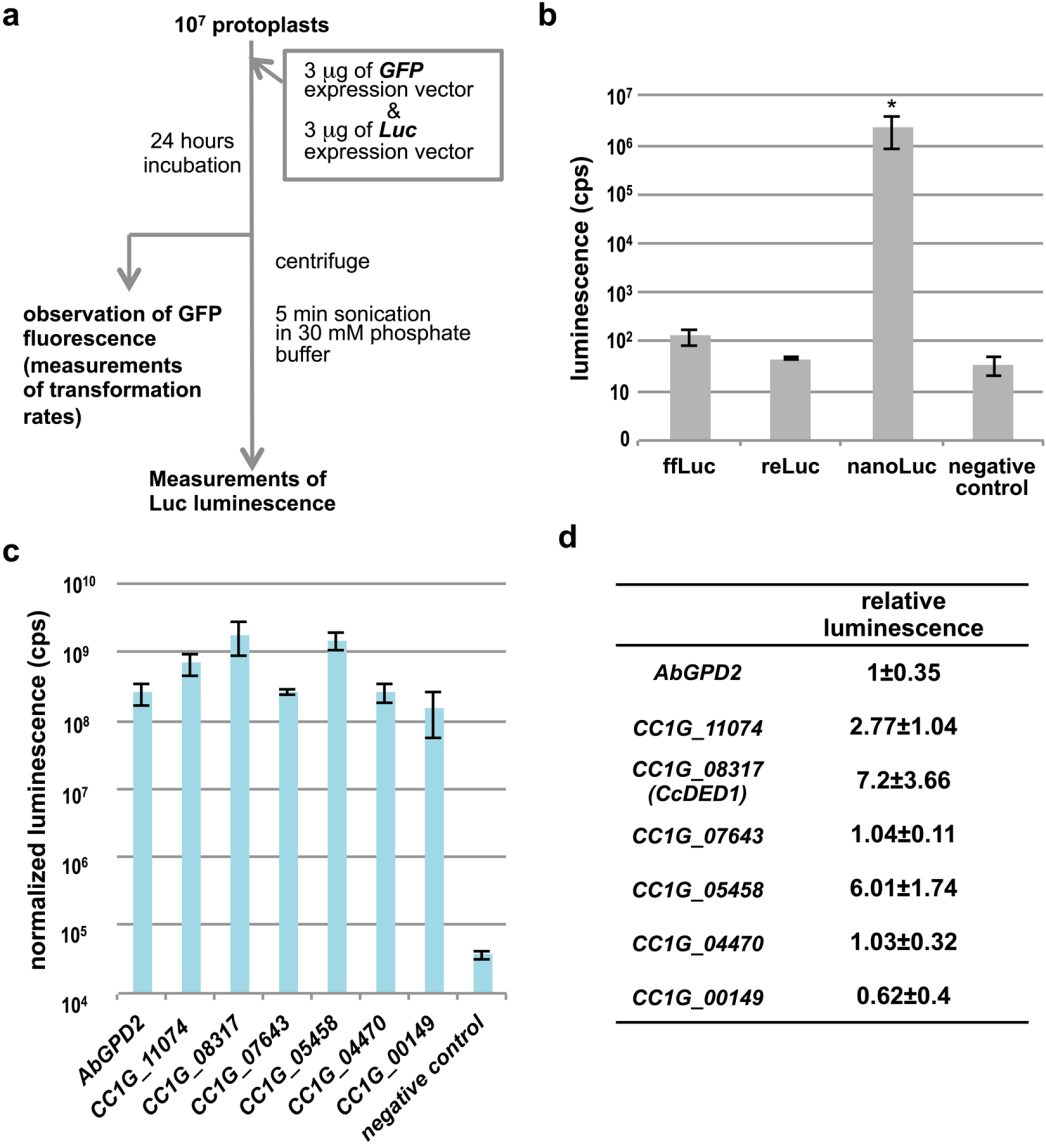
NanoLuc-based luciferase assay using cryopreserved protoplasts identified novel constitutive active promoters in *C. cinerea*. (**a**) The flow of luciferase assay in *C. cinerea*, corresponding data are given in Supplementary Fig. 3. (**b**) Comparison of three different luciferases driven by *AbGPD2pro*. Asterisk shows statistical difference calculated with Turkey’s HSD test (*P* < 0.05). (**c**) Comparison of activities of various promoters fused with *NanoLuc* luciferease. Luminescences of NanoLuc were normalized by transformation rate. Bars: S.D., n = 3. (**d**) Relative luminescence results of the experiments shown in (**c**), with the luminescence of *AbGPD2pro* defined as 1. Number ± S.E.

Fewer strong promoters are available for use in mushroom-forming basidiomycetes compared to yeast or other model filamentous fungi^7^. Data from transcriptomic studies using RNA-seq and qRT-PCR had suggested potential candidates for constitutively active promoters in mushroom-forming basidiomycetes^20^. Because of the limited evaluation methods able to detect promoter activity *in vivo*, only a few constitutively active promoters from mushroom-forming basidiomycetes have been reported^21^. Accordingly, we established a luciferase-based promoter-reporter assay in *C. cinerea* to search for constitutively active promoters with high expression. In a transient assay using 10^7^ cryopreserved protoplasts, we first tested several types of luciferases from firefly, renilla and luminous shrimp (NanoLuc^22^) (Fig. 2a). Firefly luciferase had no activity in the transient assay, consistent with a previous report^7^ (Fig. 2b). Unexpectedly, the activity of renilla luciferase was also indistinguishable from that of the negative control. Only NanoLuc exhibited luminescence 24 hours after transformation (Fig. 2b). These data indicated that quantitative evaluation of promoter activity in oidia protoplasts could be achieved in *C*. *cinerea* using NanoLuc. Since the two other luciferases were not functional, dual-luciferase-assay-based normalization cannot be used in *C. cinerea*; thus, we normalized the luciferase activity by transformation rate, as determined by co-transformation with a *GFP* expression plasmid (see Methods).

### Luciferase assay identified novel promoters with strong expression in *C. cinerea*

Based on our new luciferase assay system, we next analyzed the activities of novel promoters from *C. cinerea*. We selected six candidate genes which include the presumably constitutive active gene, histone H4, and subjected them to promoter-reporter analysis (Supplementary Table 1). A 2-kbp region from upstream of the start codon of the six candidate genes was fused to the 1^st^ intron of *GPD2* from *A. bisporus* ^23^ and the *NanoLuc* coding sequence. Each reporter vector was co-transfected to protoplasts together with the *GFP* expression plasmid for normalization. All the promoter-reporter experiments were done using protoplast expressing reporters transiently (See Methods). After 24 hours of transfection, the luciferase activity in the transformed protoplasts was measured. Three of the six promoters tested showed higher activity compared with the *AbGPD2*_*pro*_—a conventional constitutively active promoter in *C. cinerea* (Fig. 2c Supplementary Fig. 3). In particular, the promoter of *CC1G_08317* displayed an activity about seven times higher than that of *AbGPD2*_*pro*_ (Fig. 2d). The *CC1G_08317* promoter fused with the fluorescent protein *Venus* also showed strong fluorescence compared with *AbGPD2*_*pro*_ (Supplementary Fig. 4). Vegetative mycelium of *CC1G_08317*_*pro*_-*Venus* transformants also showed constitutive Venus fluorescence *in vivo* (Supplementary Fig. 5). However, according to the expression pattern in the previous study^5^, *CC1G_08317* was not so strongly expressed in 13 developmental stages compared with *GPD2* or *β-tubulin* (Supplementary Fig. 6). Taken together, the NanoLuc-based promoter-reporter assay can thus be used for evaluation of promoter activity in protoplasts of *C. cinerea*. *CC1G_08317* is also annotated as *CcDED1*—a gene encoding the *C. cinerea* ortholog of ATP-dependent RNA helicase *DED1* in *Saccharomyces cerevisiae*—. Our data show that *CcDED1*_*pro*_ is a novel strongly expressing promoter in protoplasts of *C. cinerea*.

### Construction of CRISPR/Cas9 system using the novel *CcDED1* promoter

**Figure 3:**
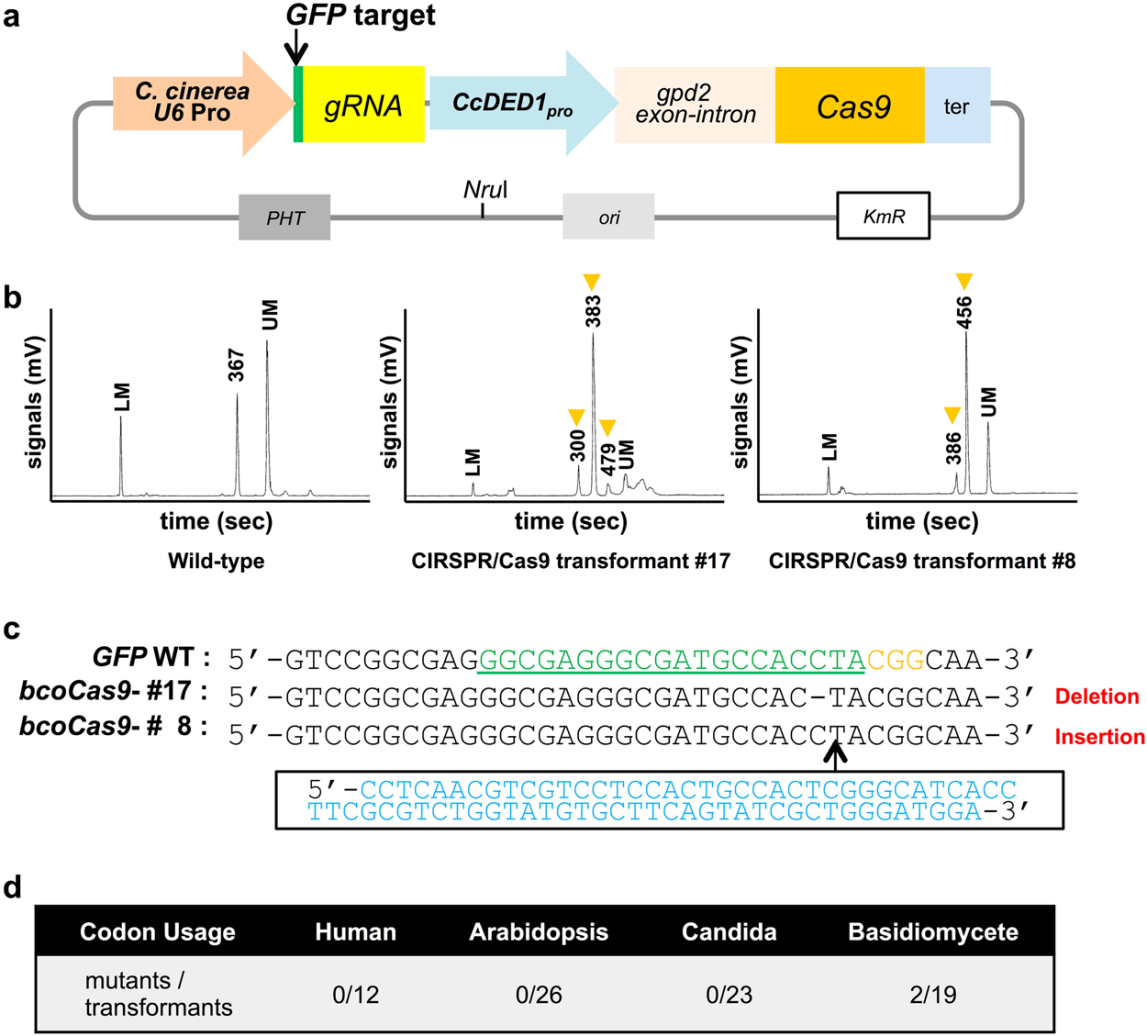
Construction of a CRISPR/Cas9 system in *C. cinerea*. (**a**) The CRISPR/Cas9 vector for *C. cinerea* genome editing. Four types of codon-optimized *Cas9* were tested. An NruI site was used for linearization. (**b**) Capillary gel electrophoresis of the PCR products of CRISPR/Cas9 transformants #17 and #8. Arrowheads show peaks not detected in wild-type. LM; lower marker, UM; upper marker. (**c**) Sequences obtained by direct sequencing of PCR products from the two CRISPR/Cas9 transformants. The target sequence of gRNA (green) and PAM sequence (yellow) are indicated. The 75-bp insertion in the line #8 is in blue. (**d**) Comparison of the mutation rates of four types of codon optimization in the transformants as detected by capillary gel electrophoresis analyses.

No CRISPR/Cas9 system has yet been reported in mushroom-forming basidiomycetes. Therefore, we constructed a CRISPR/Cas9 system optimized for *C. cinerea* using our new strong promoter, and characterized CRISPR/Cas9 constructs harboring the new promoter “*CcDED1*_*pro*_” (Fig. 3a). A *U6-snRNA* promoter from the *C. cinerea* genome was used for expression of the *gRNA*. Codon optimization is also an important issue in the optimization of CRISPR/Cas9 systems^15^, thus we tested four different codon-optimized *Cas9*: human codon- (*hco_Cas9*), Arabidopsis codon- (*Atco_Cas9*), *Candida* codon- (*caco_Cas9*), and basidiomycete codon-optimized *Cas9* (*bco_Cas9*, see Methods). As a target for CRISPR/Cas9, we constructed a CRISPR/Cas9 vector with the gRNA targeted to the sequence of the *GFP* gene^24^. We transformed cryopreserved *C. cinerea* protoplasts of a stable *GFP* expression line with the CRISPR/Cas9 vectors. The transformants were selected on medium containing hygromycin-B (Supplementary Fig. 7). Next, we analyzed the PCR-amplified genomic fragment of target sites from transformants (Fig. 3b). There were no obvious mobility changes in the PCR products from transformants expressing *Atco_Cas9*, *hco_Cas9* or *caco*_*Cas9* (data not shown). On the other hand, direct sequencing with PCR products from transformants expressing *bco*_*Cas9* showed mosaic peaks in some lines (Supplementary Fig. 8). Based on the sequencing analysis, mutation isolation efficiency was 21% (four mosaic lines per 19 transformants). In four putative genome-edited lines, two samples displaying apparently different mobility shift patterns compared to wild-type PCR products (Fig. 3b) were subjected to re-culture and direct sequencing again. The analyses revealed clear mutations: a 75-bp insertion and 1-bp deletion, respectively (Fig. 3c). The positions of these mutations were 3-bp upstream of the protospacer adjacent motif (PAM), where DSB by Cas9 was expected to have occurred^13^. These mutations clearly indicated that genome editing events by CRISPR/Cas9 had indeed occurred in these transformants.

### Isolation of genome editing lines of *C. cinerea*

**Figure 4:**
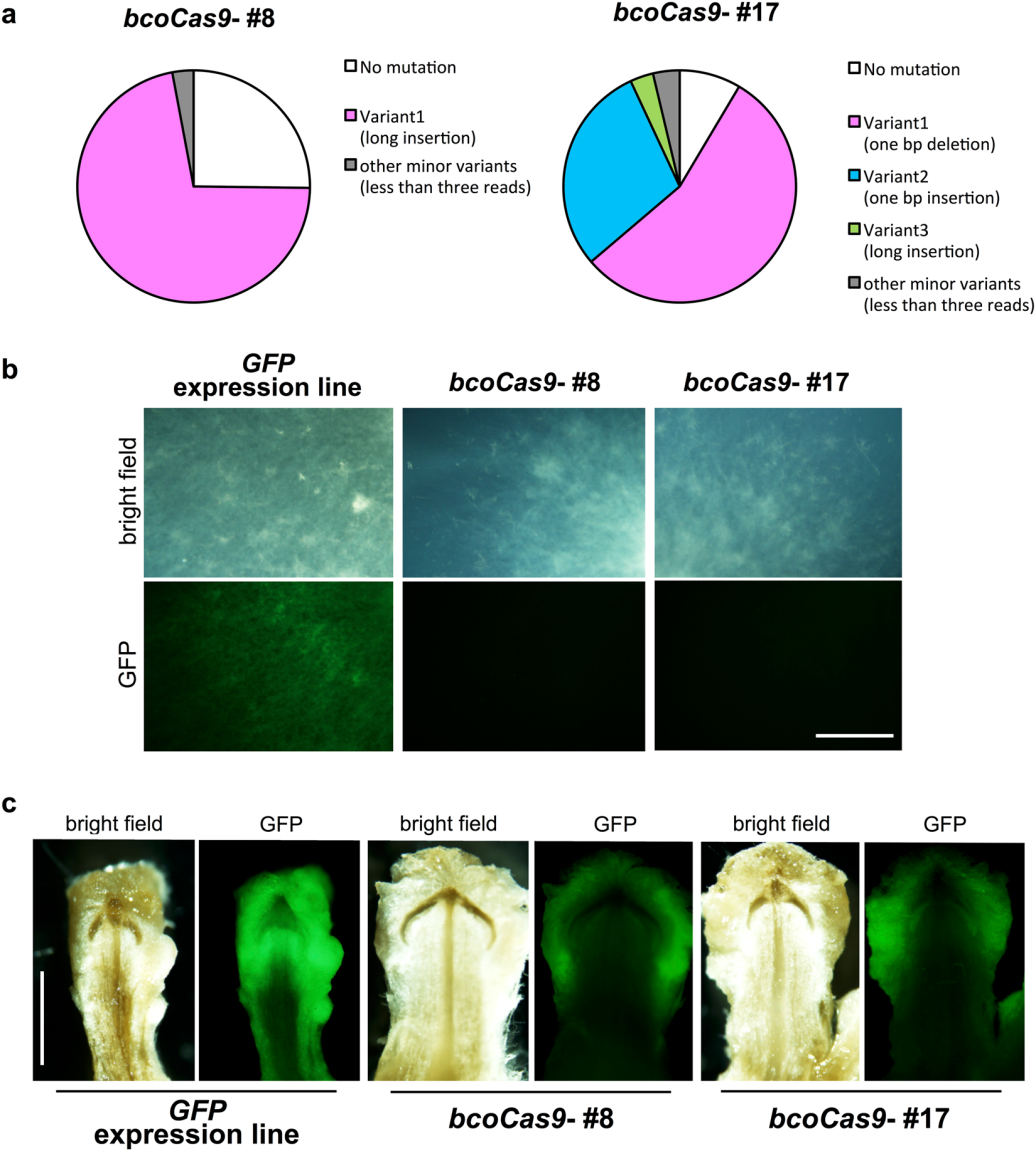
Isolation of genome-edited mushrooms. (**a**) Analyses of mosaic rates in the *C. cinerea* CRISPR/Cas9 mutant lines. Ratios of reads with mutation to total reads are described for each variant. Total read counts were 373 reads for #8 and 621 reads for #17, respectively. (**b,c**) GFP loss-of-function in the *C. cinerea* CRISPR/Cas9 mutants. GFP fluorescence was diminished in vegetative mycelium (**b**), and fruiting body primordia (**c**) in the transformants. Bars = 1 mm.

To analyze mosaicism of the two mutants, we conducted sequencing by Miseq with low depth. The deep sequencing analysis showed that 74.7% and 91.4%, respectively, of the reads in the two transformants had mutations (Fig. 4a), and the variety of mutations was very low in each mutant. Line #8, which was shown to have the 75 bp insertion in direct sequencing analysis (Fig. 3c), did not harbor any other mutations. The other line #17, which was shown to have a 1-bp deletion mutation, had only three types of mutations in the target site (Fig. 4a). Since the mosaicism of transformants could be derived from single transformed cell, genome editing events seem to have been occurred in the various timing of the development of *C. cinerea*. Since all the detected variations of mutations would cause a frame-shift in, or amino acid insertions to, *GFP*, these data suggested that both mutant lines would lead to disruption of *GFP* function. As expected, the two lines showed loss of GFP signal in hyphae and even in fruiting body primordia (Fig. 4b,c). We confirmed that the GFP expression cassette was existed on the genome from both original GFP and mutant lines (Supplementary Fig. 9). These data suggested that the two lines were genome-edited individuals. Taken together, the results showed that the efficiency of our newly constructed *bcoCas9*-based CRISPR/Cas9 system is sufficient to isolate mutants of genes of interest.

## Discussion

The throughput of reverse genetics is greatly affected by the efficiency of basic transformation techniques. In our study, a buffer consisting of 0.5 M sorbitol and 10% DMSO enabled us to preserve protoplasts prepared from *C. cinerea* oidia that can be stored at −80 °C for up to 1 year with transformation competency, although it is possible that the efficiency could be decreased after 1-year-strotage (Supplementary Fig. 1 and Fig. 1d). Mannitol has commonly been used as an osmotic stabilizer in *C. cinerea*; however, it is known that mannitol crystallizes at low temperature more easily than sorbitol^25^. It is consistent with the report that sorbitol could be used as an osmotic stabilizer for cryopreservation of protoplasts of *Schizophyllum commune* ^10^. The cryopreservation of protoplasts strengthens efforts to develop high-throughput screening in mushroom-forming basidiomycetes. In practice, we developed the NanoLuc-based reporter assay by throughput screening of more than 50 assay conditions. The repeated comparison of the activities of a number of promoters would be difficult in mushroom-forming basidiomycetes if only freshly prepared protoplasts were used. Our reporter assay used only protoplasts from oidia of *C. cinerea*; however, it might be possible that protoplasts from other tissues and species were cryopreserved by similar methods. Luciferase is one of the most suitable systems for reporter assays because of its broad dynamic range. This study is the first to combine the use of NanoLuc and cryopreserved protoplasts, establishing the luciferase assay in mushroom-forming basidiomycetes. Unexpectedly, two of three luciferases tested had no activity in *C. cinerea* (Fig. 2b). NanoLuc has 51% GC content sequence, on the other hand, firefly Luc and renilla Luc have GC contents of 47 and 37%, respectively. Since the GC content of the *C. cinerea* genome has been reported as about 51%, these data imply that codon usage could affect activity in *C. cinerea*. This is consistent with the report that AT-Rich region of foregin *hph* gene might lead truncation of its transcripts in the basidiomycete, *S. commune* ^26^. Another possibility might be the mislocalization of firefly Luc and renilla Luc in *C. cinerea*. The NanoLuc system, in contrast, performed well in our system. NanoLuc will be useful in analyzing *cis*-elements in the many genes that have been cloned in the long history of studying the genetics of mushroom-forming basidiomycetes^27, 28^. For instance, *cis*- analyses of *PriA* ^29^, a marker gene for early development of the fruiting body, whose UTR elements has been analyzed^30^, will be informative in helping to unravel some aspects of the mechanism of the onset of fruiting body development.

In this study, we optimized the CRISPR/Cas9 for mushroom-forming basidiomycetes using the constitutively active promoter *CcDED1*_*pro*_ for *Cas9* expression. *CcDED1*_*pro*_ was identified from *C. cinerea* using a NanoLuc-based reporter assay as a valuable tool for over-expression of transgenes in protoplasts. Interestingly, from *in silico* analyses, *CcDED1* presumably was not so strongly expressed compared with *GPD2* or *β-tubulin* (Supplementary Fig. 6). These data did not correspond to the results of the luciferase assay (Fig. 2). We cloned the 2-kbp upstream region of *CcDED1*, and it is possible that the activity of the 2-kb promoter might not reflect to its native transcription activity. In addition, there is the possibility that the promoter activity on the ectopically integrated transgene locus differs from its native transcription activity and expression pattern. In the CRISPR/Cas9 system, GC content might also affect the activity of Cas9 in *C. cinerea*. Only *bcoCas9*, which most resembled the *C. cinerea* genome in GC content among the codon variants of *Cas9* tested, showed genome editing activity *in vivo*. These data suggest that codon optimization for other organisms strongly affected the activity of Cas9. The unidentified subcellular localization mechanisms in *C. cinerea* also might affect the activity of Cas9.

This study is the first to report CRISPR/Cas9-based genome editing in *C. cinerea*. In the two types of mutation detected by direct sequencing analyses (Fig. 3c), the inserted 75-bp DNA fragment in the target *GFP* loci of the line #8 perfectly matched the sequence of the *C. cinerea* genome. The matched sequence corresponded to the gene *CC1G_06233*, encoding the protein phosphatase methylesterase 1. The fragment of 75 bp likely had microhomology to the targeting site (Supplementary Fig. 10). This suggests that a homology-dependent repair pathway could function in *C. cinerea* when DSBs occur. Mutant isolation efficiency, which is defined by the ratio of isolated genome-edited transformants to all transformants, was 10.5% (two out of 19 transformants). The mutant isolation efficiency can be applied for reverse genetics; however, compared with CRISPR/Cas9 efficiency in other filamentous fungi, the rate was relatively low^14–19^. To improve the efficiency, screening of other candidate *U6* promoters for *gRNA* expressions, and oidia-specific promoters for *Cas9* expression would be future targets.

In conclusion, a high-throughput transformation system, and a CRISPR/Cas9 system customized for *C. cinerea* were developed in this study. Using the CRISPR/Cas9 system, high-throughput screening to identify key genes regulating processes such as fruiting body development and the generation of useful metabolites will be possible. Our study provides tools to accelerate not only functional genetics in the model mushroom-forming basidiomycetes *C. cinerea* but also molecular breeding of various edible mushrooms.

## Methods

### Materials and culture conditions

*Coprinopsis cinerea* strain #326 (*A43mut B43mut pab1-1*) was cultured on Malt extract/Yeast extract/Glucose (MYG) medium solidified with 1.5% agar^31^. Vegetative mycelia of *C. cinerea* was grown at 28 °C (12 hrs/12 hrs light/dark) in a chamber KCLP-1400II CT (Nippon Medical & Chemical Instruments).

### Vector construction

Cloning DNA fragments of key components into the vector backbone plasmid was done by using either the standard ligation with T4 DNA ligase (NEB) or an In-Fusion HD-Cloning kit (Clontech). The GFP expression cassette composed of the *GPD2* promoter from *Agaricus bisporus*, *EGFP* (Clontech) and the heat shock protein 26 kDa gene terminator from *A. bisporus* (T26), was synthesized (GenScript) and cloned into pUC57 to yield pCop007. The wild-type *PAB1* from *C. cinerea* ^32^ was cloned into Bluescript KS(+) (Stratagene) to yield KS(+)PAB1. Both pCop007 and KS(+)PAB1 were used for establishment of the GFP-expressing *C. cinerea* line.

NanoLuc™ luciferase (Promega) vectors were constructed as follows: the NanoLuc ORF was amplified by PCR with primers described in Supplementary Table 2.

The NanoLuc fragment was cloned into the *Xba* I-*Sac* I site of pCop003 to yield pCop067. Several promoter fragments from *C. cinerea* were amplified using *C. cinerea* #326 genomic DNA as a template. The PCR products were cloned into the *Asc* I- *Nco* I site of pCop067.

The *Cas9* ORF codon-optimized for basidiomycetes was synthesized (GeneScript). The *Cas9*, gRNA and hygromycin resistance gene cassettes were introduced into plasmid pE33 modified from pDONR221(P3-P2) (Thermo Scientific) to yield the CRISPR/Cas9 vector pCop108 (see Supplemental Methods for details). The annealed oligo DNA fragment corresponding to the GFP gRNA target was inserted into the *Bsa* I site between the *U6-1pr* *o* and the gRNA scaffold sequence to yield pCop108_GFP. Restriction enzymes used in the construction were purchased from NEB. The primers and oligos for the vector construction are listed in Supplementary Table 2.

### Cryopreservation

Protoplasts were prepared as described previously^8^. For cryopreservation, 10 µl of protoplast solution suspended in MM buffer (0.5 M mannitol, 0.05 M maleate, pH 5.5) was mixed with 90 μl of preservation buffers. Protoplast solution in preservation buffer was transferred to a deep freezer (−80 °C) with/without a freezing container (Mr. Frosty; Thermo Scientific) for gradual cooling. Components of preservation buffers were as follows; Buffer 1: 0.5 M sorbitol, 0.04 M maleate, pH 5.5, 8% glycerol, Buffer 2: MM buffer (pH 5.5), 8% glycerol, Buffer 3: 0.5 M sorbitol, 0.04 M maleate, pH 5.5, Buffer 4: MM buffer (pH 5.5), 8% glycerol, Buffer 5: 0.5 M sorbitol, 0.02 M maleate, pH 5.5, 10% DMSO, 25% w/v PEG, 0.6% w/v CaCl_2_.

### PEG-based transformation

Transformation was performed as described previously^11^. Briefly, 10^8^ cells in 100 µL of protoplast preservation buffer were mixed with 10 µl of DNA solution containing 1–3 µg plasmids. The mixed solution was added to PEG-Ca buffer (25% w/v PEG and 0.6% w/v CaCl_2_) and put on ice for 20 min. A further 1 mL of PEG-Ca was added to the sample, which was stood for 5 min at room temperature. 2 mL of STC buffer was then added and the samples stored for 24 hrs. The hygromycin selection of transformed cells was performed as described by Cummings *et al*.^33^. Transformed protoplasts were spread on regeneration agar containing 0.1 mM *p*-aminobenzoic acid. After 17-18 hrs pre-incubation without hygromycin, 5 ml regeneration top-agar containing 600 mg/L hygromycin-B and transformants were sub-cultured onto minimal medium agar plates containing 100 mg/L hygromycin-B. Transformation rates were calculated by the number of hygromycin-B resistant colonies in 10^8^ preserved cells which were transformed with the PHT1 plasmid^7^.

### Transient assay of GFP fluorescence

The PEG-based transformation assay described above was conducted with 10^7^ protoplasts in a final volume of 300 µl. 10^7^ cells were sufficient to detect GFP signals in transient assay and also useful to test multiple conditions. The transformed cells were transferred to a 96-well glass bottom dish (Thermo Scientific) and cultured at 28 °C overnight. The cells were inspected using a desktop fluorescent microscope JULI (Ruskinn). The transformation rates were calculated by relating the number of GFP positive protoplasts to all the protoplasts in a field of view. Counting of GFP positive cells and counting all the cells was achieved using the macro of ImageJ^34^ (http://imagej.nih.gov/ij/).

### Luciferase assay

10^7^ protoplasts were co-transformed with 3 µg of the *NanoLuc* vector and 3 µg of the *GFP* vector pCop007. Transformed protoplasts were incubated at 28 °C for 16–24 hrs. After incubation, protoplasts were centrifuged for 5 min at 600 *g*. Pellets were re-suspended in 100 µl of phosphate buffer (pH 5.5), mixed by pipetting, and disrupted by 10 time cycles of the 30 sec-sonication with the Bioruptor sonicator (CosmoBio) with HIGH setting/the 30 sec-cooling down on ice. The cell lysates were transferred to OptiPlate-96 (PerkinElmer), and the Luc activity quantified with a Nano-Glo Luciferase assay kit (Promega) following the manufacturer’s protocol. Luminescence was measured with a luminometer GloMax®-Multi Detection System (Promega). Normalized luminescence of each sample was calculated by dividing the luminescence by the transformation rate as following.

$$normalized\,luminescence=\frac{{\rm{Luminascence}}\,{\rm{measured}}\,{\rm{by}}\,{\rm{GloMax}}}{{\rm{Number}}\,{\rm{of}}\,{\rm{GFP}}\,{\rm{positive}}\,{\rm{cells}}\,{\rm{in}}\,{\rm{unit}}\,{\rm{area}}}$$

### Isolation of genome edited lines of *C. cinerea*

Protoplasts from GFP-expressing *C. cinerea* were transformed with pCop108_GFP. After 2–3 days incubation, a part of each individual transformed mycelium was picked up and subjected to DNA extraction. The samples were directly dipped in 100 µl TE and heated at 95 °C for 10 min. One µl of the extracts was used as the template for PCR using ExTaq polymerase (Takara) following the manufacturer’s protocol. The PCR products were analyzed by capillary based electrophoresis using MultiNA (Shimazu). The samples were also subjected to direct sequencing using an ABI 3130 Genetic Analyzer (Applied Biosystems), followed by deep sequencing. GFP activity was monitored using a fluorescence stereo microscope Leica M205 FA (Leica Microsystems). The primers used for genotyping are listed in Supplementary Table 1.

### Deep sequencing analysis

The PCR products of the target site in each transformant were subjected to additional amplification with adaptor-tagged primers (GFP seqF + adapter: 5′-ACACTCTTTCCCTACACGACGCTCTTCCGATCTCGTAAACGGCCACAAGTTCAG-3′ and GFP seqR + adapter: 5′-GTGACTGGAGTTCAGACGTGTGCTCTTCCGATCTCACGAGGGTGGGCCAG-3′). The library was denatured with 1 N NaOH and mixed with a PhiX control (Illumina) prior to analysis with Miseq (Illumina).The reads with mutations were categorized using the 20 bp sequence including the putative DSB site. The mosaic rate of the transgenic lines described in Fig. 4 was calculated with Excel (Microsoft).

### Statistical analysis

Statistical analyses were carried out with R (http://www.r-project.org/). The statistical significance (*P* value) was used to compare samples. The *P* values of the comparison of multiple samples were computed using Bartlett test and Kruskal-Wallis test following Turkey’s HSD test.

## Electronic supplementary material

Supplementary tables and figures
