# Supplementary material for: Genome editing in the mushroom-forming basidiomycete Coprinopsis cinerea, optimized by a high-throughput transformation system
Source: Sci Rep. 2017 Apr 28;7:1260. doi: 10.1038/s41598-017-00883-5 (PMC5430836; doi:10.1038/s41598-017-00883-5)
Supplement: Supplementary file 1 — Supplementary tables and figures [file 41598_2017_883_MOESM1_ESM.pdf]

Title:

Genome editing in the mushroom-forming basidiomycete *Coprinopsis cinerea,* optimized by a high-throughput transformation system

Authors:

Shigeo S. Sugano[1*], Hiroko Suzuki[1*], Eisuke Shimokita[1, 2], Hirofumi Chiba[1,3], Sumihare Noji[1], Yuriko Osakabe[1,3], Keishi Osakabe[1, 3]

Affiliations:

[1] Center for Collaboration among Agriculture, Industry, and Commerce, Tokushima University, Japan

[2] Tokushima Prefectural Agriculture, Forestry and Fisheries Technology Support Center, Tokushima Prefecture, Japan

[3] Faculty of Bioscience and Bioindustry, Tokushima University, Japan

* These authors contributed equally to this work; Shigeo S. Sugano & Hiroko Suzuki

Corresponding Author

Keishi Osakabe

Faculty of Bioscience and Bioindustry, Tokushima University, Japan

Tel: +81-88- 634-6418

kosakabe@tokushima-u.ac.jp

Supplementary methods

Construction of CRISPR/Cas9 vector

pDONR221(P3-P2) (Thermo Scientific) was used as the backbone vector for the CRISPR/Cas9 vector. Multi-cloning sites; *Apa* I, *Nhe* I, *Hin*d III, *Eco*R I, *Asc* I, *Nco* I, *Sac* I, *Sna*B I, and *Pac* I were introduced into the *Apa* I- *Eco*R V site of pDONR221(P3-P2) to yield pE33.

The Cas9 ORF codon-optimized for basidiomycetes was synthesized (GeneScript), assembled, and cloned into the *Nco* I-*Sac* I site of pE33-bcoCas9. To create the Cas9 expression cassette, the *CcDED1* gene promoter (Pded1), and the heat shock protein 26kDa gene terminator from *Agaricus bisporus* (T26) were amplified. PCR fragments, Pded1 and T26 fragments were cloned into the *Asc* I-*Nco* I site and the *Sac* I-*Pac* I site of pE33-bcoCas9, respectively, to yield pE33-Pded1bcoCas9T26. The sgRNA expression cassette composed of the endogenous putative U6 snRNA gene promoter (U6-1p) from *C. cinerea* and sgRNA scaffold, was synthesized (GeneScript) and cloned into the *Hin*d III-*Asc* I site of pE33-Pgdp2coCas9T26 to yield pE33_U6gRNA-Pded1bcoCas9T26. The hygromycin resistance marker gene driven by the beta-tubulin gene promoter and terminator were amplified from pPHT1[29] as a template and cloned into the *Apa* I site of pE33_U6gRNA_Pded1bcoCas9T26 to yield pCop108. The annealed oligo DNA corresponding to the GFP gRNA target was inserted into the *Bsa* I site between the U6-1p and the gRNA scaffold sequence to yield pCop108_GFP. More details and the plasmids shown above are available upon request.

Supplementary Table 1. The six genes whose promoter activity was analyzed by luciferase assay.

Gene ID	Annotation
CC1G_11074	ubiquitin ligase
CC1G_08317	ATP-dependent RNA helicase DED1
CC1G_07643	Ser/Thr Protein Kinsase
CC1G_05458	Histone H4
CC1G_04470	endopeptidase
CC1G_00149	ADP-ribose

Supplementary Table 2. The primer list used in this study.

Purpose	Name	Sequence
fireflyLuc cloning	XbaI_ffLuc_Fw	5’-TCCAGGTCAAAGCATCTAGAATTACACGGCGATCTTTCCG -3’
	SacI_ffLuc_Rv	5’-CGAGGAGCTACTAAGAGCTCTTACGACATTTGTTCATTTTTGAGAAC -3’
reLuc cloning	XbaI_reLuc_Fw	5’-TCCAGGTCAAAGCATCTAGAATGACTTCGAAAGTTTATGATCCAG-3’
	SacI_reLuc_Rv	5’-CGAGGAGCTACTAAGAGCTCATGGAAGACGCCAAAAACATAAAG -3’
NanoLuc cloning	XbaI_nanoLuc_Fw	5’-CAGGTCAAAGCATCTAGAATGGTCTTCACACTCGAA-3’
	SacI_nanoLuc_Rv	5’-AGGAGCTACTAAGAGCTCTTACGCCAGAATG-3’
cloning of constitutive active promoter candidates		
CC1G_11074pro cloning	Pro1 Fw	5’-TTCCGCGGTGGCGCGCCTAACAACGTCAACGCAGTCGAG-3’
	Pro1 Rv	5’-GTACTGACCGCCATGGGTCTACGAGTTGGATGGGAG-3’
CC1G_08317pro cloning	Pro2 Fw	5’-TTCCGCGGTGGCGCGCCTAACAACGTCAACGCAGTCGAG-3’
	Pro2 Rv	5’-GTACTGACCGCCATGGGTCTACGAGTTGGATGGGAG-3’
CC1G_07643pro cloning	Pro3 Fw	5’-TTCCGCGGTGGCGCGCCGACGCTCGATGCCCATGTTCAAC-3’
	Pro3 Rv	5’-GTACTGACCGCCATGGGGTGAATCACTGGGCGAGG-3’
CC1G_05459pro cloning	Pro4 Fw	5’-TTCCGCGGTGGCGCGCCATCTACCAAGTCCAGCTCGATG-3’
	Pro4 Rv	5’-GTACTGACCGCCATGGCTTATTCTAAGTTTGTTGGG-3’
CC1G_04470pro cloning	Pro5 Fw	5’-TTCCGCGGTGGCGCGCCTACCATCACCATCTATCTCTC-3’
	Pro5 Rv	5’-GTACTGACCGCCATGGGGTAGTGGTTCAGGAGAGAG-3’
CC1G_00149pro cloing	Pro6 Fw	5’-TTCCGCGGTGGCGCGCCCTTTACCCAAGCTCGTTCCTG-3’
	Pro6 Rv	5’-GTACTGACCGCCATGGTGTGATAGGCGATGTGGGATTTG-3’
U6 snRNA promoter cloning	CcU6_Fw	5’-CCGCGGTGGCGCGCCGTCGTCAAAGGGAGAAA -3’
	CcU6_Rv	5’-CCTCTCTCGGTCTCCAATCACCATTGAATTTG -3’
caco_Cas9 cloning	caCas9Fw	5’-GTCAAAGCATCTAGAATGGATAAAAAGTAT-3’
	caCas9Rv	5’-AGCTACTAAGAGCTCAGCCCCTTCATCACC-3’
hco_Cas9 cloning	hCas9Fw	5’-TCAACAAATCTTCAACTAATCCATGGCGGTCAGTACACCA -3’
	hCas9Rv	5’-GGGGATAAACTTAATTAAGGtacgtaTTGAACAGCATTGCGTT -3’
Atco_Cas9 cloning	AtcoCas9Fw	5’-TTCGCTTCCAGGTCAAAGCATCTAGAGATAAGAAGTACTCTATCGGA -3’
	AtcoCas9Rv	5’-GGGATAAACTTAATTAAGGtacgtaTTGAACAGCATTGCGTT-3’
gRNA for GFP target	GFPT1_F	5’-GATTGGGCGAGGGCGATGCCACCTA -3’
	GFPT1_R	5’-AAACTAGGTGGCATCGCCCTCGCC -3’
Venus cloning	Venus_in fusion_Fw	5' -GGTCAAAGCATCTAGA GTGAGCAAGGGCGAGGA-3'
	Venus_in fusion_Rv	5' -AGGAGCTACTAAGAGCTCTTATCGTCCATGCCGAGA- 3'
genotyping of the target sequence of GFP	GFP genotyping_Fw	5’-CCATGGGTGAGCAAGGGCGAGG-3’
	GFP genotyping_Rv	5’-TTAATTAATCGCCCTCGAACTTCAC-3'

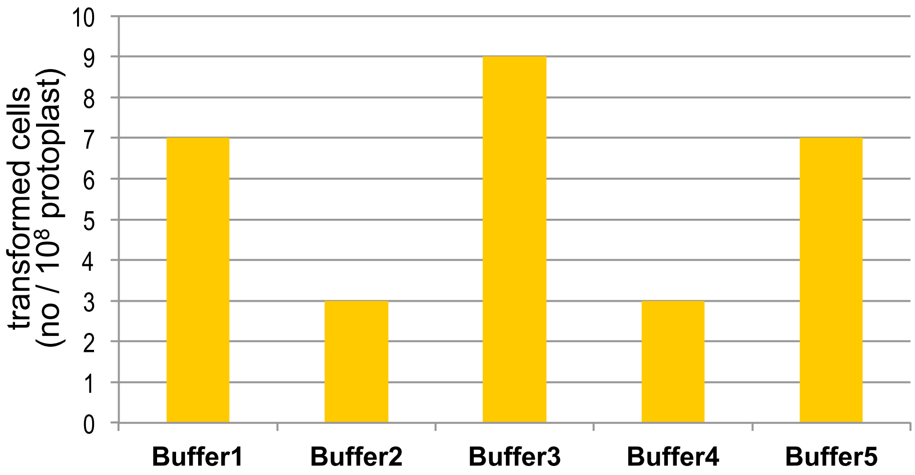

Supplementary Fig. 1. Transformation competency of one-year cryopreserved protoplasts of *C. cinerea.*

Survival rates and transformation rates of protoplasts stored in -80 ºC for one year were tested. Transformed cell numbers in 10^8 protoplasts stored in one year. Each buffers were corresponding to that in Fig. 1b.

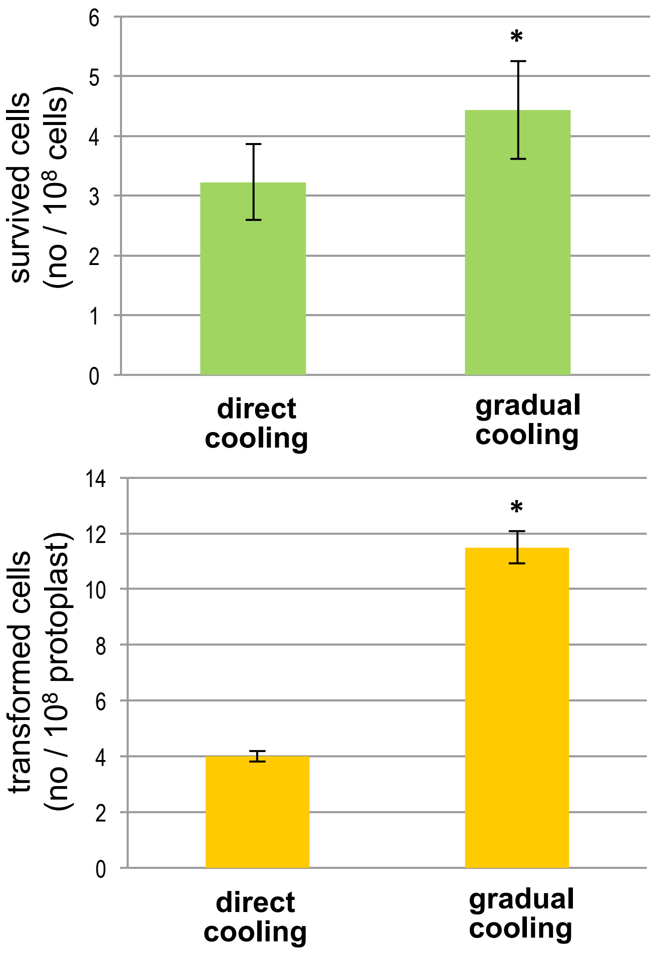

Supplementary Fig. 2. Effects of gradual cooling for cryopreservation of protoplasts

Survival rates and transformation rates of protoplasts frozen with different cooling rates were assessed. a) Survival cell numbers in 10^8 protoplasts stored with direct cooling in –80 ºC or gradual cooling at –1 ºC/min to –80 ºC b) Transformed cell numbers in 10^8 protoplasts stored with direct cooling in –80 ºC or gradual cooling with –1 ºC/min to –80 ºC. These protoplasts, prepared with 0.6 M sorbitol and 10% DMSO buffer, were stored at –80 ºC for 3 weeks. Bar = S.D. n = 3. Asterisks show statistical significance (*t*-test, $P < 0.05$).

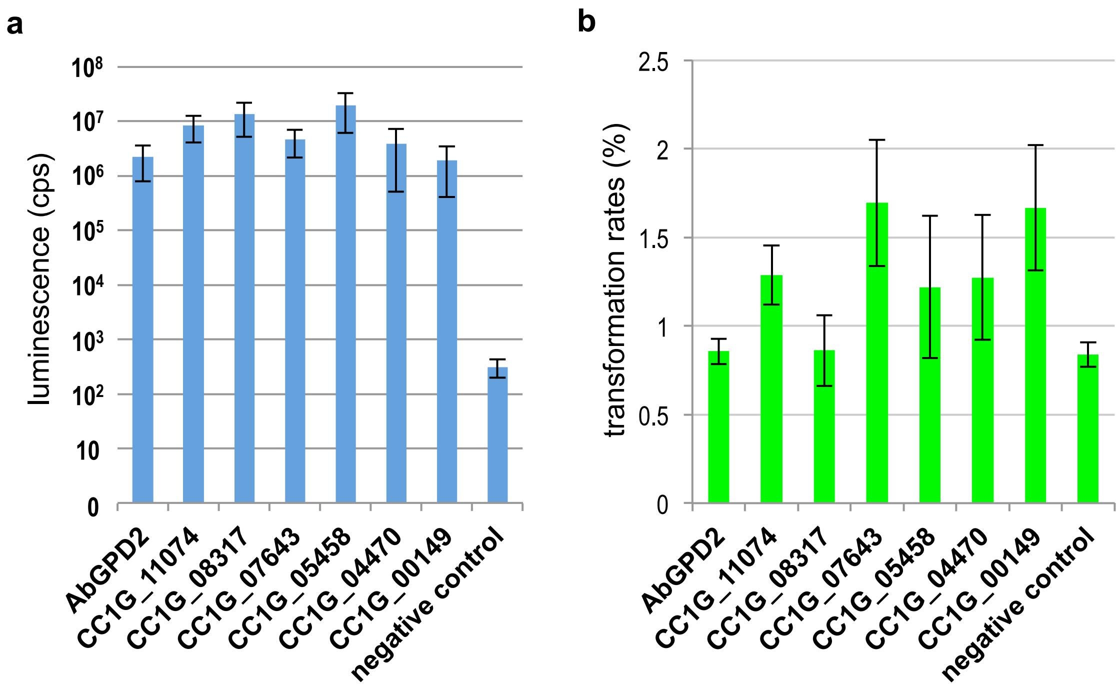

Supplementary Fig. 3. NanoLuc fluorescence and transformation rates in Fig. 2c. Luminescence (a), and transient transformation rates detected by GFP fluorescence (b) were analyzed in the same sample in Fig. 2c. Normalized luminescence in Fig. 2c was the luminescence divided by transient transformation rates. Error bars show S.E., n = 3.

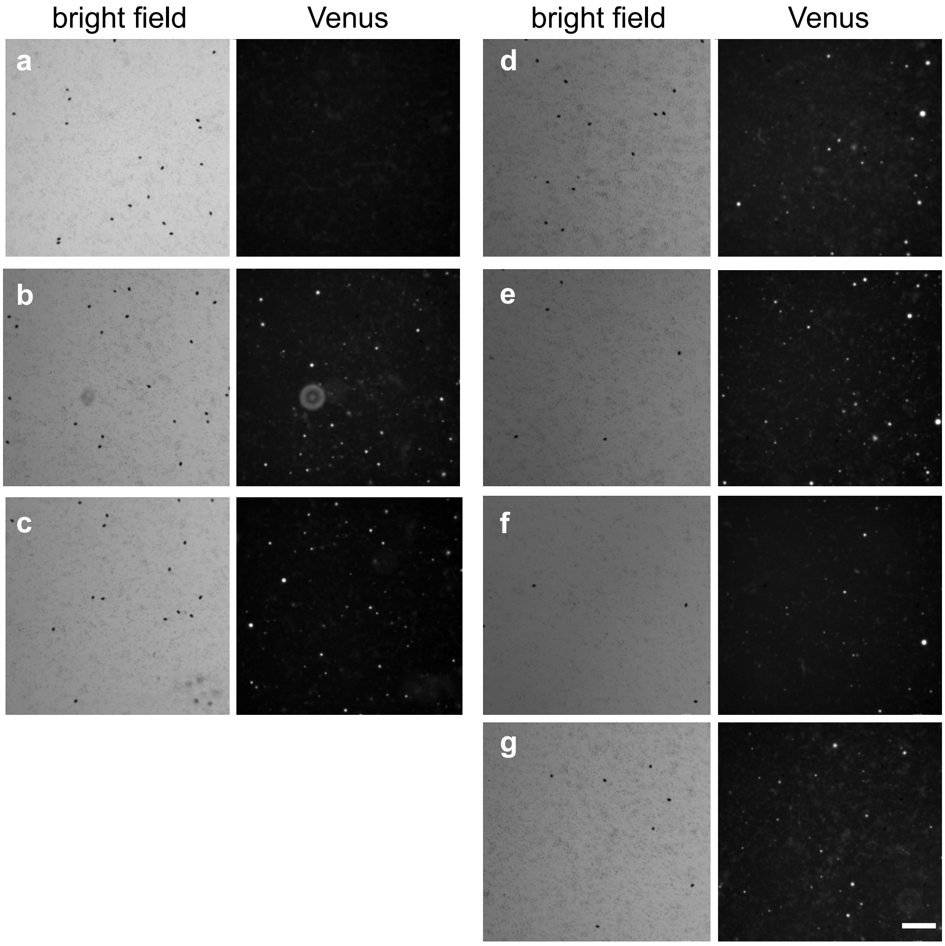

Supplementary Fig. 4. **Fluorescence based transient assay of the promoters used in luciferase assays.**

Representative images of oidia protoplasts 24 hours after transformation. Transformants harboring the *Venus* gene driven by the each promoter. a: *AbGPD2pro*, b: *CC1G_11074pro*, c: *CC1G_08317* (*CcDED1*) *pro,* d: *CC1G_07643pro,* e: *CC1G_05458pro,* f: *CC1G_04470pro*, e: *CC1G_00149pro.* The fluorescent images were acquired with the same exposure time. Bar = 100 µm.

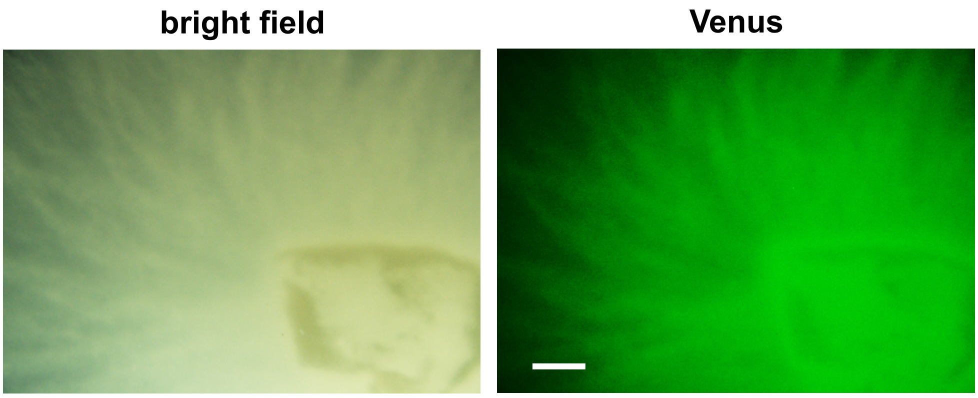

Supplementary Fig. 5. Venus fluorescence of a CcDED1pro::Venus stable transformant.
Two-day-old vegetative mycelium of the stable transformant of *CcDED1pro::Venus*. Bar = 1 mm

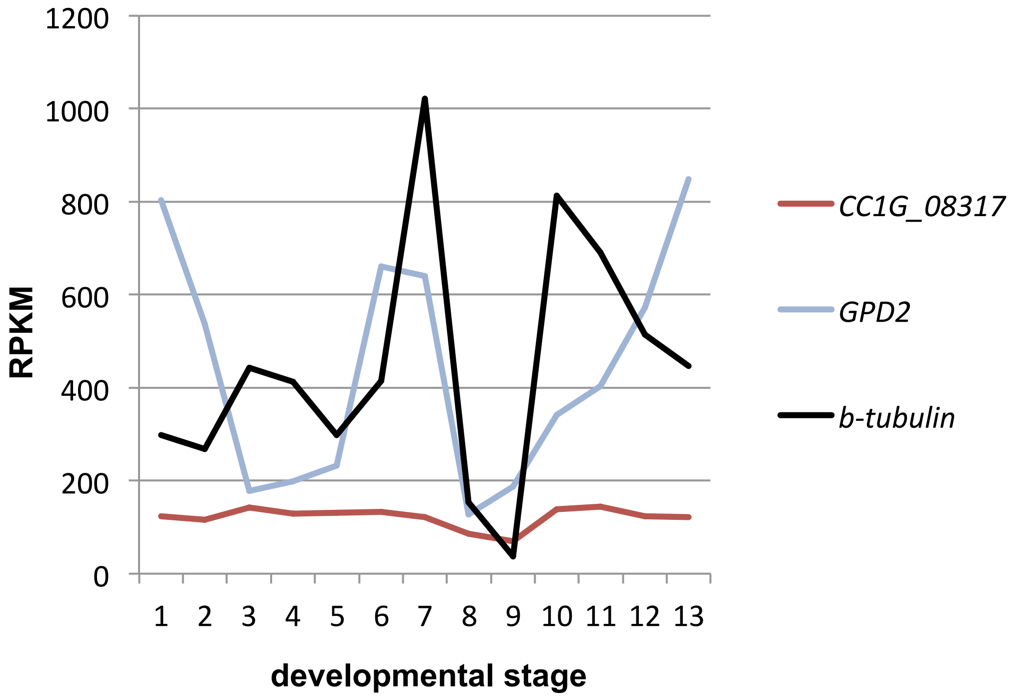

Supplementary Fig. 6. Expression levels of *CcDED1* and other constitutive active genes.

Vertical axis indicates RNA expression levels. Horizontal axis indicates developmental stages identified in Muraguchi et al. PLoS ONE, 2015. 1: vegetative mycelium, 2: hyphal knot 3: small primodia 4: fruiting body primodia at 0 hr, 5: fruiting body primodia at 12 hr, 6: cap at 24 hr after the trigger light, 7: stipe at 24 hr after the trigger light, 8: cap at 30 hr after the trigger light, 9: stipe at 24 hr after the trigger light, 10: cap at 36 hr after the trigger light, 11: stipe at 36 hr after the trigger light, 12: cap at 39 hr after the trigger light, 13: stipe at 39 hr after the trigger light

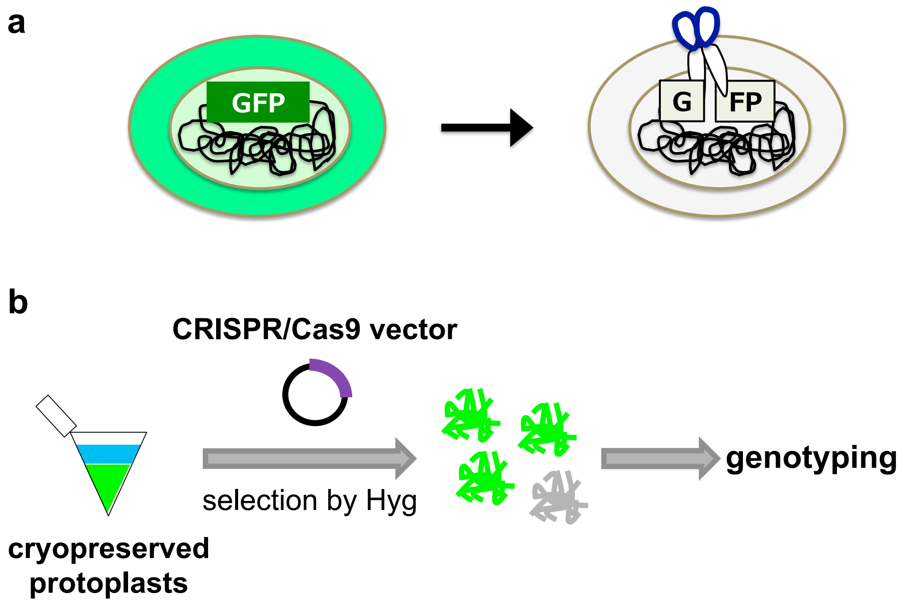

Supplementary Fig. 7. Schematic image of CRISPR/Cas9-based genome editing in *C. cinerea*

(a) Schematic image of genome editing experiments. If the target sequence of *GFP* transgene is broken, GFP fluorescence of the cells is diminished. (b) Flow of screening of genome editing in mushroom. A CRISPR/Cas9 vector targeting GFP sequence was transformed to cryopreserved protoplasts of *GFP* expressing lines and selected with hygromycin-B. Selected colonies were incubated for 2 days in MYG medium and subjected to genotyping by PCR

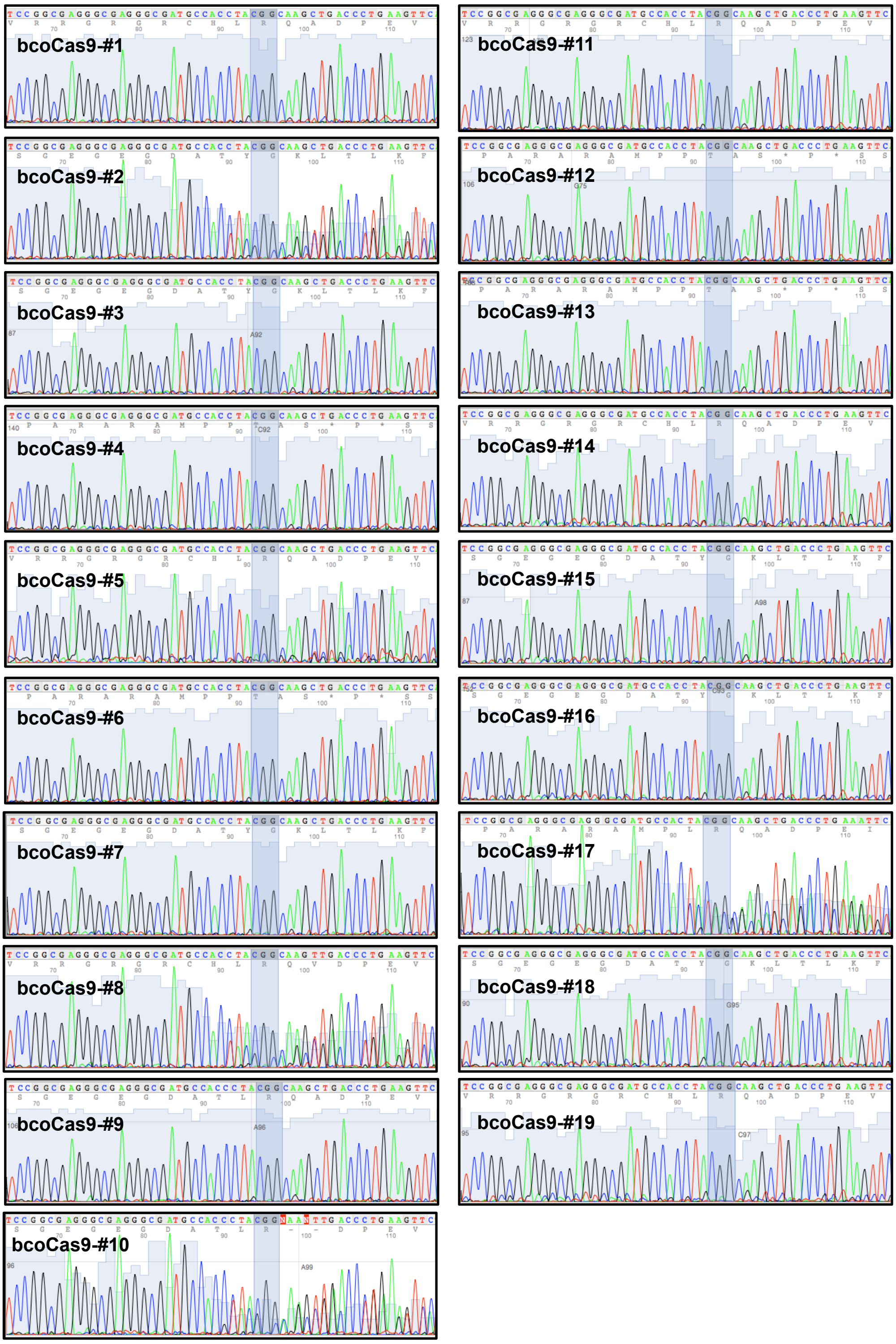

Supplementary Fig. 8. Direct sequencing analysis of transgenic lines with *bcoCas9* . Line # was attached on the upper left part on each panel. PAM sequence was highlighted.

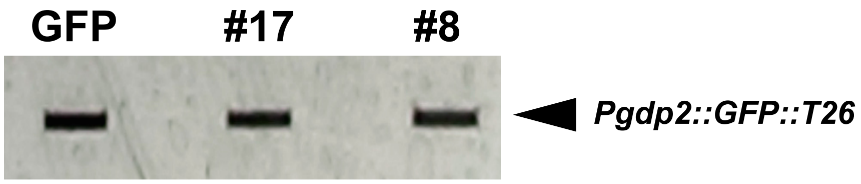

Supplementary Fig. 9. Genomic PCR to identify the GFP expression cassette in transgenic lines #8 and #17.
Genomic PCR was performed with genomic DNA from the original GFP line and transgenic lines with *bcoCas9* (Lines #8 and #17).
To amplify entire the *GFP* expression cassette, primers Pgdp2_H3F2 (5'-GCCAAGCTTAAGAGGTCCGCAAGTAGATT-3') and T26_EcoR2 (5'-ATGGAATTCTTGAACAGCATTGCGTTATTTAT-3') were used. GFP: the original line, #17: transgenic line #17, #8: transgenic line #8.

```
CC1G_06233   :CAGCCACTCCAGTAGGGCGCCGTACTCCTCAACGTCGTCCTCCACTGCCACTCGGGCATCACCTTCGCGTCTGGTATGTGCTTCAGTATCGCTGGGATGGATACTCTTG
GFP          :---------GCGAGGGCGATGCCACC---------------------------------------------------------------------------TACGGCAA
```

Supplementary Fig. 10. Multiple alignment of the genomic DNA sequence of *CC1G_06233* and gRNA target sequence of *GFP*.
75bp inserted sequence in bcoCas9 -#8 line was colored with blue. And a putative microhomology sequences were colored in red. PAM sequence was underlined.
